# Artificial intelligence and classification of mature lymphoid neoplasms

**DOI:** 10.37349/etat.2024.00221

**Published:** 2024-04-23

**Authors:** Joaquim Carreras, Rifat Hamoudi, Naoya Nakamura

**Affiliations:** University of Campania “L. Vanvitelli”, Italy; ^1^Department of Pathology, Tokai University School of Medicine, Isehara 259-1193, Japan; ^2^Department of Clinical Sciences, College of Medicine, University of Sharjah, Sharjah P.O. Box 27272, United Arab Emirates; ^3^Division of Surgery and Interventional Science, University College London, WC1E 6BT London, UK

**Keywords:** Artificial intelligence, machine learning, deep learning, artificial neural networks, non-Hodgkin lymphomas, pan-cancer series, prognosis, gene expression

## Abstract

Hematologists, geneticists, and clinicians came to a multidisciplinary agreement on the classification of lymphoid neoplasms that combines clinical features, histological characteristics, immunophenotype, and molecular pathology analyses. The current classification includes the World Health Organization (WHO) Classification of tumours of haematopoietic and lymphoid tissues revised 4th edition, the International Consensus Classification (ICC) of mature lymphoid neoplasms (report from the Clinical Advisory Committee 2022), and the 5th edition of the proposed WHO Classification of haematolymphoid tumours (lymphoid neoplasms, WHO-HAEM5). This article revises the recent advances in the classification of mature lymphoid neoplasms. Artificial intelligence (AI) has advanced rapidly recently, and its role in medicine is becoming more important as AI integrates computer science and datasets to make predictions or classifications based on complex input data. Summarizing previous research, it is described how several machine learning and neural networks can predict the prognosis of the patients, and classified mature B-cell neoplasms. In addition, new analysis predicted lymphoma subtypes using cell-of-origin markers that hematopathologists use in the clinical routine, including *CD3, CD5*, *CD19*, *CD79A*, *MS4A1* (*CD20*), *MME* (*CD10*), *BCL6*, *IRF4* (*MUM-1*), *BCL2*, *SOX11*, *MNDA*, and *FCRL4* (*IRTA1*). In conclusion, although most categories are similar in both classifications, there are also conceptual differences and differences in the diagnostic criteria for some diseases. It is expected that AI will be incorporated into the lymphoma classification as another bioinformatics tool.

## Introduction

This manuscript provides a brief communication of the current status of the classification of mature lymphoid neoplasm and aims to relate it to the development of prediction and classification tools using artificial intelligence (AI). First, a summary of the current lymphoma classification is made. The current classification is the revised 4th edition of the World Health Organization (WHO) classification of tumours of hematopoietic and lymphoid tissues. This classification has recently been updated and evolved into the International Consensus Classification (ICC) and the proposed 5th edition of the WHO Classification of haematolymphoid tumours (WHO-HAEM5). A comparison between both classifications on mature lymphoid neoplasms is made. Secondly, the applications of AI in the assessment of the prognosis and classification of mature lymphoid neoplasms are shown. This section summarizes part of previous research in this field. Finally, we propose a simple method based on a neural network to classify mature B-cell neoplasms using conventional cell-of-origin markers.

## Classification of mature lymphoid neoplasms

### Evolution of lymphoma classification

There have been several lymphoma classifications, including the Rappaport Classification, the Rye Classification for Hodgkin Disease, the Kiel Classification for Lymphoma, the Working Formulation for Non-Hodgkin Lymphoma (HL), the Revised European American Lymphoma Classification (REAL), and the WHO Classification.

The current lymphoma classification goes back to 1994 when the revised Europe an American classification of lymphoid neoplasms (REAL) was released [1]. This classification was revised in 1997, and the approach was improved by including morphological features, immunophenotype, clinical characteristics, and molecular pathology methods in addition to histological assessment [2]. The classification changed under the WHO umbrella in 2001, 2008, and 2016 [3–5].

The current classification, known as the 2016 revision of the WHO Classification of lymphoid neoplasms, was developed by hematopathologists, geneticists, and physicians by agreement. It contains both updated and preliminary entities for various hematological neoplasia [5]. This classification is currently scheduled to be changed in 2022–2023. In the summer of 2022, the Clinical Advisory Committee reported the ICC of mature lymphoid neoplasms [6], which was followed by the ICC of myeloid neoplasms and acute leukemias [7], commentary of precision medicine [8], and genomic profiling for clinical decision making in lymphoid neoplasms [9]. Almost concomitantly, an overview of the upcoming WHO-HAEM5 focusing on lymphoid, myeloid, and histiocytic/dendritic neoplasms was published as well [10–12]. Because the 5th edition of the WHO Classification is based on the previous version, both the ICC and the 5th WHO are very similar.

### Relevant subtypes of mature B-cell neoplasms

The mature lymphoid and histiocytic/dendritic cell neoplasms are classified into different groups: mature B-cell neoplasms, classic HL, mature T-cell and natural killer (NK)-cell neoplasms, immunodeficiency-associated lymphoproliferative disorders, and histiocytic and dendritic cell neoplasms [6].

The most “relevant subtypes” of mature B-cell neoplasms, which are well known by general pathologists, are the following: chronic lymphocytic leukemia (CLL)/small lymphocytic lymphoma (SLL), splenic marginal zone lymphoma (MZL), hairy cell leukemia, lymphoplasmacytic lymphoma (LPL) and Waldenstrom macroglobulinemia, multiple myeloma, extranodal MZL of mucosa-associated lymphoid tissue (MALT) lymphoma, nodal MZL, follicular lymphoma (FL), mantle cell lymphoma (MCL), diffuse large B-cell lymphoma (DLBCL), plasmablastic lymphoma, human herpesvirus 8 (HHV-8)-positive DLBCL not otherwise specified (NOS), primary effusion lymphoma, Burkitt lymphoma (BL), high-grade B-cell lymphoma (HBCL), and primary mediastinal large B-cell lymphoma (LBCL), among others [6].

These “relevant” entities are present both in the 2022 ICC and WHO-HAEM5 [10]. A comparison between the ICC of mature lymphoid neoplasms [6], the WHO-HAEM5 [10], and the WHO Classification, revised 4th edition [5], with a focus on the mature B-cell neoplasms is shown in Table 1. Overall, the classifications are comparable as they derived from the same original source, the revised 4th edition. However, although most categories are similar in both classifications, there are also conceptual differences and differences in the diagnostic criteria for some diseases. In the ICC, the changes from the 2016 WHO Classification are highlighted with an asterisk.

**Table 1:** Comparison between ICC 2022, WHO-HAEM5, and WHO revised 4th edition

**The ICC of Mature Lymphoid Neoplasms: a report from the Clinical Advisory Committee (2022)**	**WHO Classification, 5th edition (2022), mature B-cell neoplasms**	**WHO Classification, revised 4th edition**
**-**	**Pre-neoplastic and neoplastic small lymphocytic proliferations**	**-**
Monoclonal B-cell lymphocytosis (CLL type/non-CLL type)	Monoclonal B-cell lymphocytosis	Monoclonal B-cell lymphocytosis
CLL/SLL	CLL/SLL	CLL/SLL
B-cell prolymphocytic leukemia	Entity deleted	B-cell prolymphocytic leukemia
**Splenic B-cell lymphomas (BCLs) and leukemias**	**Splenic BCLs and leukemias**	**-**
Hairy cell leukemia	Hairy cell leukemia	Hairy cell leukemia
Splenic MZL	Splenic MZL	Splenic MZL
Splenic diffuse red pulp small BCL	Splenic diffuse red pulp small BCL	Splenic diffuse red pulp small BCL
Hairy cell leukemia-variant	Splenic BCL/leukemia with prominent nucleoli	Not previously included (encompassing hairy cell leukemia variant and some cases of B-cell prolymphocytic leukemia)
**LPL**	**LPL**	**LPL**
LPL/Waldenstrom macroglobulinemia	LPL	LPL
**MZL**	**MZL**	**MZL**
Extranodal MZL of MALT (MALT lymphoma)	Extranodal MZL of MALT	Extranodal MZL of MALT
Primary cutaneous marginal zone lymphoproliferative disorder*	Primary cutaneous MZL	Not previously included (originally included under extranodal MZL of MALT)
Nodal MZL	Nodal MZL	Nodal MZL
Pediatric nodal MZL	Pediatric MZL	Pediatric MZL
**FL**	**FL**	**FL**
*In situ* follicular neoplasia	*In situ* follicular B-cell neoplasm	*In situ* follicular neoplasia
FL	FL	FL
Pediatric-type FL	Pediatric-type FL	Pediatric-type FL
Duodenal-type FL	Duodenal-type FL	Duodenal-type FL
Testicular FL*	-	-
BCL2 apoptosis regulator (*BCL2*)-Rearrangement-negative, CD23-positive follicle center lymphoma	-	-
**Cutaneous follicle center lymphoma**	**Cutaneous follicle center lymphoma**	-
Primary cutaneous follicle center lymphoma	Primary cutaneous follicle center lymphoma	Primary cutaneous follicle center lymphoma
**MCL**	**MCL**	**MCL**
*In situ* mantle cell neoplasia	*In situ* mantle cell neoplasm	*In situ* mantle cell neoplasia
MCL	MCL	MCL
Leukemic non-nodal MCL	Leukemic non-nodal MCL	Leukemic non-nodal MCL
-	**Transformations of indolent BCLs**	-
-	Transformations of indolent BCLs	Not previously included
**LBCLs**	**LBCLs**	**LBCLs**
DLBCL, NOS/germinal center B (GCB)-cell subtype/activated B-cell (ABC) subtype	DLBCL, NOS	DLBCL, NOS
T cell/histiocyte-rich LBCL	T-cell/histiocyte-rich LBCL	T-cell/histiocyte-rich LBCL
HBCL, with *MYC* and *BCL2* rearrangements*/HBCL with *MYC* and BCL6 transcription repressor (*BCL6*) rearrangements*	DLBCL/HBCL with *MYC* and *BCL2* rearrangements	HBCL with *MYC* and *BCL2* and/or *BCL6* rearrangements
Anaplastic lymphoma kinase (ALK)-positive LBCL	ALK-positive LBCL	ALK-positive LBCL
LBCL with interferon regulatory factor 4 (IRF4) rearrangement*	LBCL with IRF4 rearrangement	LBCL with IRF4 rearrangement
LBCL with 11q aberration*	HBCL with 11q aberrations	Burkitt-like lymphoma with 11q aberration
Nodular lymphocyte-predominant BCL	-	-
Lymphomatoid granulomatosis	Lymphomatoid granulomatosis	Lymphomatoid granulomatosis
Epstein-Barr virus–positive polymorphic B-cell lymphoproliferative disorder, NOS*	-	-
EBV-positive DLBCL, NOS	EBV-positive DLBCL	EBV-positive DLBCL, NOS
Pediatric nodal MZL	Pediatric MZL	Pediatric MZL
DLBCL associated with chronic inflammation	DLBCL associated with chronic inflammation	DLBCL associated with chronic inflammation
Fibrin-associated DLBCL	Fibrin-associated LBCL	Not previously included (previously considered a subtype of DLBCL associated with chronic inflammation)
HHV-8 and EBV-negative primary effusion-based lymphoma*	Fluid overload-associated LBCL	Not previously included
Plasmablastic lymphoma	Plasmablastic lymphoma	Plasmablastic lymphoma
Primary DLBCL of the central nervous system/primary DLBCL of the testis*	Primary LBCL of immune-privileged sites	Not previously included, encompassing primary DLBCL of the clinical nurse specialist (CNS) in revised 4th edition (plus primary LBCL of the vitreoretina and primary LBCL of the testis)
Primary cutaneous DLBCL, leg type	Primary cutaneous DLBCL, leg type	Primary cutaneous DLBCL, leg type
Intravascular LBCL	Intravascular LBCL	Intravascular LBCL
Primary mediastinal LBC	Primary mediastinal LBCL	Primary mediastinal LBCL
Mediastinal gray-zone lymphoma*	Mediastinal grey-zone lymphoma	BCL, unclassifiable, with features intermediate between DLBCL and classic HL
HBCL, NOS	HBCL, NOS	HBCL, NOS
Nodular lymphocyte predominant BCL*	-	-
**BL**	**BL**	-
BL	BL	BL
**HHV-8–associated lymphoproliferative disorders**	**Kaposi’s sarcoma-associated herpesvirus (KSHV)/HHV8-associated B-cell lymphoid proliferations and lymphomas**	-
Primary effusion lymphoma	Primary effusion lymphoma	Primary effusion lymphoma
HHV-8-positive DLBCL, NOS	KSHV/HHV8-positive DLBCL	HHV8-positive DLBCL, NOS
HHV-8-positive germinotropic lymphoproliferative disorder	KSHV/HHV8-positive germinotropic lymphoproliferative disorder	HHV8-positive germinotropic lymphoproliferative disorder
Pediatric nodal MZL	Pediatric MZL	Pediatric MZL
-	**Lymphoid proliferations and lymphomas associated with immune deficiency and dysregulation**	-
-	Hyperplasias arising in immune deficiency/dysregulation	Not previously included, encompassing non-destructive post-transplant lymphoproliferative disorders, among others
-	Polymorphic lymphoproliferative disorders arising in immune deficiency/dysregulation	Not previously included, encompassing polymorphic posttransplant lymphoproliferative disorders, other iatrogenic immunodeficiency-associated lymphoproliferative disorders, among others
EBV-positive mucocutaneous ulcer*	EBV-positive mucocutaneous ulcer	EBV-positive mucocutaneous ulcer
-	Lymphomas arising in immune deficiency/dysregulation	Not previously included, encompassing monomorphic posttransplant lymphoproliferative disorders, classic HL posttransplant lymphoproliferative disorders, lymphomas associated with human immunodeficiency virus (HIV) infection, among others
-	Inborn error of immunity-associated lymphoid proliferations and lymphomas	Lymphoproliferative diseases associated with primary immune disorders
-	**Plasma cell neoplasms and other diseases with paraproteins**	-
-	**Monoclonal gammopathies**	-
Primary cold agglutinin disease*	Cold agglutinin disease	Not previously included
Immunoglobulin M (IgM) monoclonal gammopathy of undetermined significance (MGUS)/IgM MGUS, plasma cell type*/IgM MGUS, NOS*	IgM MGUS	IgM MGUS
Non-IgM MGUS	Non-IgM MGUS	Non-IgM MGUS
-	Monoclonal gammopathy of renal significance	Not previously included
Monoclonal Ig deposition diseases/Ig light chain amyloidosis (AL)*/localized AL amyloidosis*/light chain and heavy chain deposition disease	Diseases with monoclonal Ig deposition	-
-	Ig-related AL amyloidosis	Primary amyloidosis
Monoclonal Ig deposition diseases	Monoclonal Ig deposition disease	Light chain and heavy chain deposition disease
Heavy chain diseases	Heavy chain diseases	Heavy chain diseases
Mu heavy chain disease	Mu heavy chain disease	Mu heavy chain disease
Gamma heavy chain disease	Gamma heavy chain disease	Gamma heavy chain disease
Alpha heavy chain disease	Alpha heavy chain disease	Alpha heavy chain disease
**Plasma cell neoplasms**	**Plasma cell neoplasms**	**Plasma cell neoplasms**
Solitary plasmacytoma of bone/extraosseous plasmacytoma	Plasmacytoma	Plasmacytoma
Multiple myeloma (plasma cell myeloma)	Plasma cell myeloma	Plasma cell myeloma
Multiple myeloma with recurrent genetic abnormality [cyclin D (CCND) family translocation, musculoaponeurotic fibrosarcoma (MAF) family translocation, nuclear receptor binding SET domain protein 2 (NSD2) translocation, with hyperdiploidy]	-	-
-	Plasma cell neoplasms with associated paraneoplastic syndrome	-
-	Polyneuropathy, organomegaly, endocrinopathy, M-protein, skin changes (POEMS) syndrome	-
-	TEMPI syndrome	-
-	Adenopathy and an extensive skin patch overlying a plasmacytoma (AESOP) syndrome	(Same) Except AESOP syndrome not previously included

Changes from the 2016 WHO classification in the ICC classification, based on the ICC 2022, WHO-HAEM5, and WHO revised 4th edition [1–10]. In the ICC, the changes from the 2016 WHO Classification are highlighted with an asterisk. Bold font indicates the principal lymphoma types. TEMPI: telangiectasia-erythrocytosis-monoclonal gammopathy-perinephric-fluid collections-intrapulmonary shunting syndrome; -: no data

*Note.* Adapted from “A comparison of the International Consensus and 5th World Health Organization classifications of mature B-cell lymphomas,” by Falini B, Martino G, Lazzi S. Leukemia. 2023;37:18–34 (https://www.nature.com/articles/s41375-022-01764-1). CC BY; “The 5th edition of the World Health Organization Classification of Haematolymphoid Tumours: lymphoid neoplasms,” by Alaggio R, Amador C, Anagnostopoulos I, Attygalle AD, Araujo IBO, Berti E, et al. Leukemia. 2022;36:1720–48 (https://www.nature.com/articles/s41375-022-01620-2). CC BY; “WHO classification of tumours [Internet],” Lyons: International Agency for Research on Cancer; c1965–2024 [cited 2023 Jul 7]. Available from: https://whobluebooks.iarc.who.int/structures/haematolymphoid/

Among the multiple hematological neoplasias of the classification, the worth entities are the following: hairy cell leukemia-variant (splenic BCL)/leukemia with prominent nucleoli), primary cutaneous marginal zone lymphoproliferative disorder, HBCL with *MYC* and *BCL2* rearrangements, HBCL with *MYC* and *BCL6* rearrangements, LBCL with *IRF4* rearrangement, LBCL with 11q aberration, HHV-8 and Epstein-Barr virus (EBV)-negative primary effusion-based lymphoma (fluid overload-associated LBCL), primary DLBCL of the central nervous system and testis (immune privileged sites), mediastinal gray-zone lymphoma, nodular lymphocyte predominant BCL, EBV-positive mucocutaneous ulcer, primary cold agglutinin disease, and monoclonal Ig deposition diseases (and related).

**Figure 1:**
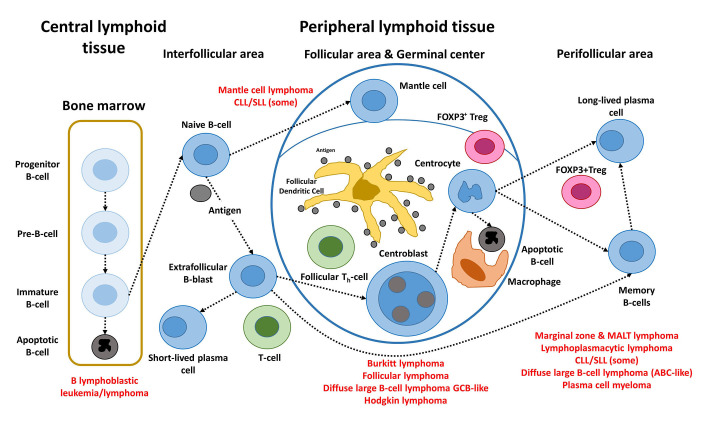
Steps of the physiological B-cell differentiation, and the relationship with the postulated cell-of-origin of the different mature BCLs. +: positive; FDC: fixed-dose combination; TFH: T-follicular helper; Pre: precursor; T_h_: T helper cell *Note*. Adapted from “Artificial intelligence predicted overall survival and classified mature B-cell neoplasms based on immuno-oncology and immune checkpoint panels,” by Carreras J, Roncador G, Hamoudi R. Cancers. 2022;14:5318 (https://www.mdpi.com/2072-6694/14/21/5318). CC BY.

The postulated origin of some of the most relevant mature B-cell neoplasms is shown in Figure 1. These non-HL subtypes correspond to various stages of B-cell differentiation. For example, FL, BL, and DLBCL originate and/or have a stage of differentiation from mature B lymphocytes of the germinal centers of lymphoid follicles. In FL, the most characteristic molecular change of FL, is the *IGH/BCL2* translocation t(14; 18)(q32; q21) that occurs in the bone marrow of the patients. Later, when the B lymphocytes recirculate within the germinal center, secondary changes occur and lymphoma develops [5, 13–15].

B-cell neoplasms recapitulate several phases of B-cell development, according to the WHO classification of tumours of hematopoietic and lymphoid tissues, updated 4th edition 2016 [5]. MCL is caused by pre-germinal B lymphocytes, which are peripheral B cells of the mantle zone; however, some are of post-germinal origin [16, 17]. FL, BL, and DLBCL develop from GCB lymphocytes. The B lymphocytes of the germinal centers are centrocytes and centroblasts, and are intermingled by numerous follicular T-helper cells, follicular dendritic cells, macrophages, and regulatory T lymphocytes (Treg) [Fork head box P3 (FOXP3)-positive Tregs]. Proliferation, apoptosis, somatic hypermutation (SHM), and Ig switch class recombination (SCR) all occur in germinal centers. BCL of the marginal zone develops during the post-germinal center development stage. Other lymphoma subtypes to mention are CLL/SLL, whose normal counterpart is CD5 molecule (CD5)-positive B lymphocytes with mutated or unmutated Ig heavy variable (*IGHV*) genes [16], and LPL, which arises from post-follicular B cells that differentiate into plasma cells [5]. Similarly, plasma cell myeloma is caused by long-lived plasma cells from the post-germinal center [5]. MALT lymphoma (extranodal MZL of MALT) derives from post-germinal center marginal-zone B cells [5, 18, 19]. Finally, when *MYC* and *BCL2* are rearranged, HBCL with *MYC* and *BCL2* and/or *BCL6* rearrangements emerge from mature GCB cells, although this is questionable in *MYC* and *BCL6* instances [5].

### Highlights in diffuse large B-cell lymphoma and FL

The changes, i.e., highlights, in the ICI 2022 classification of aggressive BCLs are of special interest [6]. In this section, 14 subtypes are specified.

In DLBCL, NOS, the sub-classification based on the cell-of-origin is maintained, but molecular profiling using the 5 or 7 functional subgroups.

The Chapuy-Shipp classification identified 5 groups, C1 to C5, with coordinated genetic signatures in 304 DLBCL samples [20]. The progression-free survival was different between C0/C1/C4 (associated with favorable prognosis), *versus* C2 (intermediate prognosis), and *versus* C3/C5 (poor prognosis). The C0 cluster was characterized by absence of molecular changes. The C1 cluster was characterized by *BCL6* structural variants (SVs). Cluster C2 by tumor protein 53 (*TP53*) mutations. The C3 cluster was characterized by *BCL2* mutations and SVs that result in a juxtaposition of *BCL2* to Ig heavy chain (*IgH*) enhancer. The C3 cluster also had frequent mutations of lysine methyltransferase 2D (*KMT2D*), CREB binding protein (*CREBBP*), and enhancer of zeste 2 polycomb repressive complex 2 subunit (*EZH2*), and a cell-of-origin GCB-like. The C5 cluster was characterized by 18q and chromosome 3 gains, and mutations of *CD79B*, MYD88 innate immune signal transduction adaptor (*MYD88*), and Pim-1 proto-oncogene, serine/threonine kinase (*PIM1*), and a cell-of-origin ABC-like.

The Wright classification [21] described an algorithm that classified the patients into 7 genetic subtypes (MCD, N1, A53, BN2, ST2, EZB, MYC^+^, and MYC^–^) that aided to develop a rationally targeted therapy of DLBCL on Bruton tyrosine kinase (BTK), phosphoinositide 3-kinase (PI3K), BCL2, Janus kinase (JAK), IRF4, and EZH2 molecules. The MCD subtype was characterized by *MYD88* (L265P), and *CD79B* mutations. The BN2 by *BCL6* fusion and notch receptor 2 (*NOTCH2*) mutations. The EZB by *BCL2* fusion, and *EZH2* and *TNFRSF14* mutations. The ST2 had tet methylcytosine dioxygenase 2 (*TET2*) mutations. The A53 had *TP53*, and the N1 had *NOTCH1* mutations. Among the subtypes, the BN2 associated with a favorable survival.

Of note, the work of Lacy et al. [22] identified 6 groups including MYD88, BCL2, SOCS1/SGK1, TET2/SGK1, and NOTCH2, along with an unclassified group. These groups were comparable to the work of Runge et al. [23].

Among others, additional highlights of the ICI 2022 classification of aggressive BCLs were that LBCL with an 11q aberration entity is still considered provisional, and now it is closer to DLBCL than BL. Nodular lymphocyte-predominant BCL is different from classic HL, and closer to T-cell histiocyte-rich LBCL. The primary DLBCL of the testis is recognized as a specific subtype, such as DLBCL of the central nervous system. HHV-8 and Eptein-Barr virus-negative primary effusion-based lymphoma is a provisional entity. EBV-positive mucocutaneous ulcer changed from a provisional to a definitive entity. And HBCL with MYC rearrangement is divided into cases with *MYC* and *BCL2*, and *MYC* and *BCL6* rearrangements. Of note, in the WHO-HAEM5, the BCL6 rearrangement becomes “less relevant”, and cases with *MYC* and *BCL6* rearrangements are classified as DLBCL, NOS, or HGBL, NOS based on the cytomorphological features.

Regarding FL, the difference between the ICI 2022 and WHO-HAEM5 is that in HAEM5 the grading is no longer mandatory in cases of classic FL, and two new FL subtypes are defined, the follicular LBCL (FLBL, the previous FL grade 3B), and FL with uncommon features (uFL). Conversely, the ICI 2022 retained the morphologic grading (grades 1–2, 3A, and 3B).

## Applications of AI in the classification and prognosis of B lymphoid neoplasms

### Strong and weak AI

According to McCarthy, AI is “the science and engineering of making intelligent machines, particularly intelligent computer programs” [24], and it is a field that combines computer science and data tools to solve problems. It includes both machine-learning and deep-learning techniques. AI is a useful tool for analyzing large amounts of data to make predictions and classifications. AI is divided into two categories: “weak AI” and “strong AI” [25].

Strong AI, also known as artificial general intelligence (AGI), should be indistinguishable from human intelligence; therefore, it is currently a theoretical concept that must pass the Turing test [26]. Strong AI would solve various problems (security, entertainment, and content creation, as well as behavioral recognition and prediction), eventually teaching itself to solve new ones [25, 27]. In contrast, weak AI (sometimes known as “narrow”) focuses on restricted sorts of tasks. Weak AI requires humans to give the learning algorithm settings and necessary training data to solve issues accurately [25, 27].

### Machine learning and artificial neural networks

In our research, the AI analyses included several machine learning techniques and artificial neural networks. The machine learning included the C5 algorithm for decision tree, Bayesian network, classification and regression (C & R) tree, Chi-squared (*χ*^2^) automatic interaction detection (CHAID) tree, discriminant analysis, nearest neighbor analysis [k-Nearest Neighbor (KNN)], logistic regression, linear support vector machine (LSVM), quick, unbiased, efficient statistical tree (QUEST), random forest, random trees, SVM, tree-AS, and eXtreme Gradient Boosting (XGBoost) linear and tree, among others. The artificial neural networks comprised multilayer perceptron (MLP) and radial basis functions.

The description of the different techniques is present in the original publications [28–37]. The C5 decision tree predicts only categorical variables; it is characterized as being robust when there is missing data or the model includes a large number of predictors, the training time is relatively short, and has a simple interpretation.

The Bayesian network is a graphical model that links the different variables of a dataset (known as nodes) using arcs. For instance, a Bayesian network can be constructed to predict a specific disease based on the presence of different symptoms or data. If information is unavailable, the Bayesian networks are incredibly resilient and produce the best feasible forecast using whatever information is available [28–37].

The C & R tree node is another type of tree-based classification and prediction method that can handle missing data and large datasets effectively. Unlike the C5 tree, both the target and predictors can be continuous or categorical. The CHAID decision tree uses *χ*^2^ analysis to calculate the optimal splits. The target variable can be both continuous and categorical, such as in the C & R tree, but can create non-binary splits with more than 2 or more subgroups [28–37].

The discriminant analysis searches for group memberships based on linear combinations. Nearest Neighbor Analysis recognizes patterns of data and classifies the cases based on their resemblance to other cases. Logistic regression, also known as nominal regression, is similar to linear regression, but the target variable is categorical. LSVM is useful for analyzing large datasets with large numbers of predictors. QUEST is another method of binary classification that creates trees characterized by lower processing time than the C & R tree. The random forest is based on the bagging algorithm, and can handle large datasets and missing data, and can highlight the most relevant predictors [28–37].

### Use of machine learning and neural networks in the classification and prognostic assessment of mature B-cell neoplasms

AI has the potential to revolutionize biological research and clinical practice. We recently employed weak (narrow) AI to categorize the various subtypes of mature B lymphoid neoplasms and predict patient prognosis. The most important findings from our recent publications that used AI to classify and predict non-HLs are summarized in Table 2 [14, 31–37].

**Table 2:** Applications of AI in hematopathology research

**Summary**	**Available website address**	**Reference**
This research integrated previous studies and added a new analysis of macrophages, including three-dimensional (3D) rendering. The focus was on immuno-oncology markers.	https://doi.org/10.3390/cancers14215318	[14]
This research predicted the prognosis of FL using 120 different and independent artificial neural networks. The random number generator was used to generate the different overall survival predictions.	https://doi.org/10.3390/biomedinformatics2020017	[31]
The overall survival of MCL was predicted using two strategies. First, a dimensionality reduction was based on the previously identified genes. Second, on immuno-oncology panels. The results were correlated with the Lymphoma/Leukemia Molecular Profiling Project (LLMPP) MCL35 proliferation assay.	https://doi.org/10.3390/healthcare10010155	[32]
The overall survival and cell-of-origin molecular subtypes of DLBCL were predicted using artificial neural networks and a pan-cancer immune-oncology panel of 730 genes.	https://doi.org/10.3390/cancers13246384	[33]
A neural network predicted (classified) several non-HL subtypes, including FL, MCL, DLBCL, BL, and MZL. All the genes of the array were used and a cancer transcriptome panel. The survival of a pan-cancer series was also performed.	https://doi.org/10.3390/make3030036	[15]
This research used immunohistochemical analysis and AI to predict the survival of DLBCL, with a focus on the protein and gene expression of the colony stimulating factor 1 receptor (CSF1R).	https://doi.org/10.3390/hemato2020011	[34]
This research analyzed the predictive value of caspase-8 (CASP8) and related markers [cleaved CASP3, cleaved poly(ADP-ribose) polymerase 1 (PARP1), BCL2, TP53, MDM2, MYC, Ki67, E2F1, CDK6, MYB, LMO2, and tumor necrosis factor-alpha-induced protein 8 (TNFAIP8)] in DLBCL using immunohistochemical stainings and several machine learning and artificial neural networks.	https://doi.org/10.3390/biomedinformatics1010003	[35]
In DLBCL, several AI techniques were used for multidimensionality reduction to predict the overall survival of the patients. As a result, two markers were highlighted, programmed cell death1 ligand 1 (PD-L1/CD274) and IKAROS, which were later tested by immunohistochemistry in an independent series of cases.	https://doi.org/10.3390/ai2010008	[36]
Using a MLP and 25 genes, the overall survival of DLBCL was predicted. In the final model, the prognosis was predicted using *MYC*, *BCL2*, and enolase 3 (*ENO3*).	http://mj-med-u-tokai.com/pdf/450107.pdf	[37]

The first publication of 2020 analyzed the gene expression of 100 cases of DLBCL that were stratified according to a risk score based on the expression of *CD163* (high *versus* low), which is a marker of M2-like tumor-associated macrophages (TAMs) [37]. The statistical method was an artificial neural network (MLP). The gene expression data of 54,614 gene-probes were used as input (predictors), and the output (predicted variable) was the overall survival outcome as dead versus alive. As a result, 25 genes were highlighted. Further correlation with already known genes with predictive value in DLBCL using neural networks, gene-set enrichment analysis (GSEA), and Cox regression analysis managed to reduce the list of 25 to only three genes, *MYC* (cell cycle), *BCL2* (apoptosis), and *ENO3* (cell metabolism) [37].

The second publication improved the analysis algorithm [36]. Instead of predicting only one variable (the overall survival), the algorithm also predicted many other clinicopathological variables that had prognostic relevance in DLBCL, such as cell-of-origin molecular subtype, age, lactate dehydrogenase (LDH) ratio, Eastern Cooperative Oncology Group (ECOG) performance status, clinical stage, and extra-nodal disease (among others). As a result, the list of pathogenic genes was refined, and after several steps of dimensionality reduction, a final set of 16 genes was highlighted. The clinical relevance of these 16 genes was tested using several machine learning techniques, GSEA, functional network association analysis, and conventional overall survival analysis [36]. Then, two markers that were highlighted (*PD-L1* and *IKAROS*), were successfully validated at the protein level by immunohistochemistry in an independent series of cases from Tokai University Hospital [36].

The use of machine learning and artificial neural networks was also applied to data obtained from digital image quantification of protein levels of several markers, using immunohistochemistry in DLBCL [35]. In this project, the aim was to evaluate the prognostic value of CASP8 and to correlate with other related markers such as CASP3, cleaved PARP, BCL2, TP53, MDM2, MYC, Ki67, E2F1, CDK6, MYB, LMO2, and TNFAIP8. The results showed that high expression of CASP8 correlated with a favorable prognosis for the patients [35].

Artificial neural networks were also used to predict the subtype of non-HL using gene expression data. In this analysis, all the genes of the array or a pan-cancer panel were used to predict the lymphoma subtype with high performance. Therefore, weak AI successfully made lymphoma diagnosis without the use of histological images [15]. Of note, work on MCL was also carried out [32], and the same methodology was applied to non-tumour immunological conditions such as celiac disease and ulcerative colitis [28, 29].

**Figure 2:**
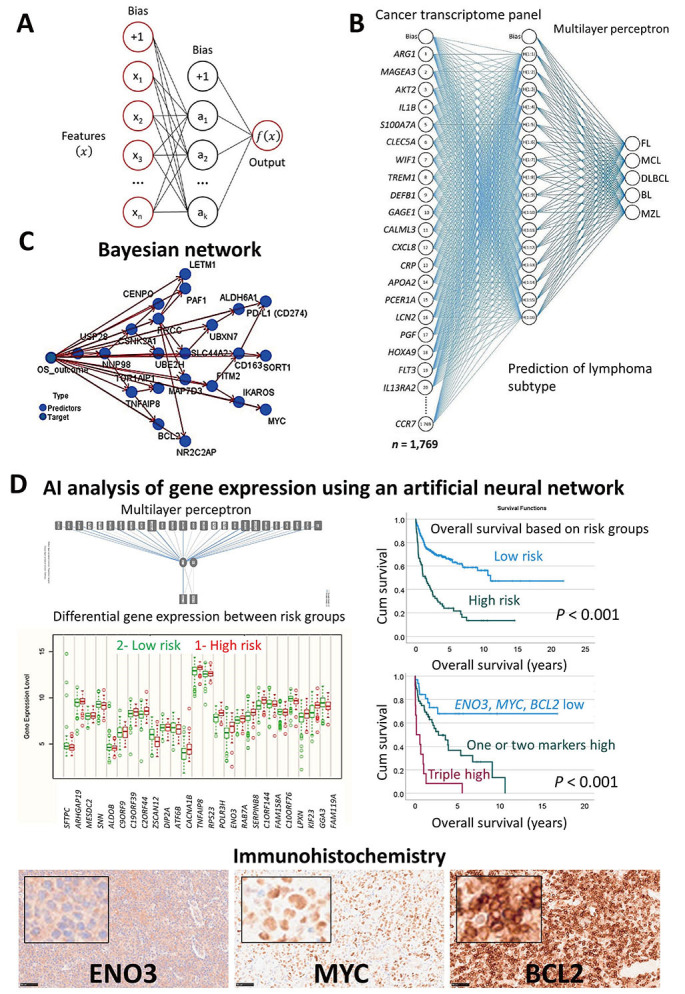
Use of AI to classify and predict non-HL. (A) Basic structure of an artificial neural network; (B) a neural network was used to predict several non-HL subtypes, FL, MCL, DLBCL, BL, and MZL using a cancer transcriptome panel; (C) a Bayesian network was used to predict the prognosis of DLBCL; (D) a neural network (MLP) was used to predict the overall survival of the patients with DLBCL using gene expression data. The immunohistochemistry of one of the most relevant markers is shown (original magnification 200×). Cum: cumulative *Note*. Adapted from “Artificial intelligence predicted overall survival and classified mature B-cell neoplasms based on immuno-oncology and immune checkpoint panels,” by Carreras J, Roncador G, Hamoudi R. Cancers. 2022;14:5318 (https://www.mdpi.com/2072-6694/14/21/5318). CC BY.

In summary, machine learning and neural networks were used to predict the overall survival of the patients of the most frequent subtypes of hematological neoplasia using gene expression data. Additionally, the gene expression was used as a predictor of different lymphoma subtypes. Figure 2 shows the prediction of several mature B lymphoid neoplasms using an artificial neural network, the overall survival outcome (dead *versus* alive) using a Bayesian network, and the patients with DLBCL using transcriptomic data that highlighted *ENO3*, *MYC*, and *BCL2* genes [15, 29–37]. AI has many applications, including the prediction of non-Hogkin lymphoma subtypes, and the prognosis of the patients. The basic structure of an artificial neural network. A neural network includes a minimum of three layers. An input layer with the predictors, a hidden layer, and an output layer (Figure 2A). A neural network was used to predict several non-HL subtypes, FL, MCL, DLBCL, BL, and MZL. The input layer included the gene expression of a cancer transcriptome panel of 1,769 genes (Figure 2B). A Bayesian network was used to predict the prognosis of DLBCL. This is a graphical model that depicts the variables (known as nodes; both predictors and targets) and their connections (referred to as links and/or arcs). Although connections between nodes are made, the arcs do not always indicate a straight cause-effect relationship. This sort of network is effective when there is missing information, and it is robust since it predicts based on whatever input is included (Figure 2C). A neural network (MLP) was used to predict the prognosis of DLBCL using gene expression data. As a result, the most relevant genes were highlighted on the basis of their normalized importance for predicting the overall survival of the patients (*n* = 25), such as aldolase, fructose-bisphosphate B (*ALDOB*), disco interacting protein 2 homolog A (*DIP2A*), *TNFAIP8*, RNA polymerase III subunit H (*POLR3H*), *ENO3*, kinesin family member 23 (*KIF23*), and *GGA3*. Using these 25 genes and a risk-score formula, the overall survival of the patients was predicted with high accuracy. The correlation with other known relevant genes showed that patients with high expression of *ENO3*, *MYC*, and *BCL2* were associated with poor prognosis. Examples of cases with high expression of ENO3, MYC, and BCL2 are shown by immunohistochemistry (Figure 2D). This figure is based in part on our previous work on AI in non-HLs.

## Lymphoma classification using cell-of-origin markers: rapid communication

Machine learning and artificial neural networks can be used to classify several mature B-cell neoplasms. Here, we propose a method of classifying lymphoma subtypes based on gene expression. The publicly available dataset GSE132929 [38] was used in this analysis. The input variables (predictors) were genes that are currently used in the diagnosis by hematopathologists at the protein level by immunohistochemistry, and that reflect the cell-of-origin and the postulated cell counterparts: *CD5* (T-cell marker), CD3 epsilon subunit of T-cell receptor complex (*CD3E*, T-cell marker), *BCL2* apoptosis regulator, *BCL6* transcription repressor (germinal center marker), *IRF4* (*MUM-1*, plasma cell differentiation), membrane metalloendopeptidase [*MME*, *CD10*, common acute lymphoblastic leukemia antigen (CALLA), germinal center marker], *CD19* (B-lymphocyte marker), membrane spanning 4-domains A1 [*MS4A1* (*CD20*, B-lymphocyte marker with role in the development and differentiation of B-cells into plasma cells)], *CD79a* molecule (*CD79A*, B lymphocyte antigen receptor complex), SRY-box transcription factor 11 (*SOX11*, transcriptional activator), myeloid cell nuclear differentiation antigen (*MNDA*, marker of myelomonocytic and marginal zone B cells), and Fc receptor-like 4 [*FCRL4* (*IRTA1*, function of memory B-cells)].

**Figure 3:**
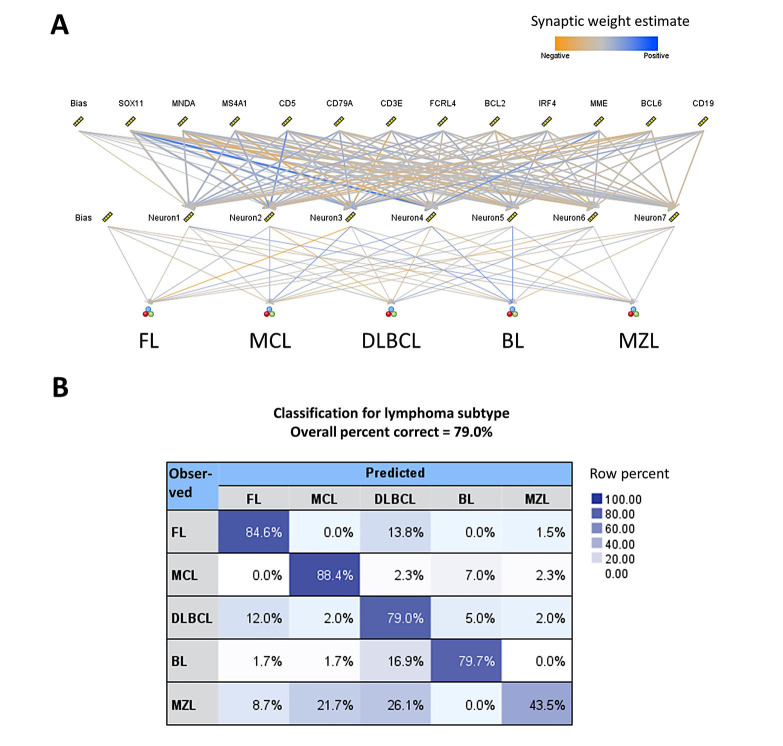
Use of AI to classify mature B-cell neoplasms using cell-of-origin markers. (A) Structure of the artificial neural network. The predictors were genes that represent several cell-of-origin markers usually used by histopathologists at lymphoma diagnosis; (B) classification matrix. The overall percentage of correct classification was 79%, being FL and MCL being the best predicted (85% and 88%, respectively)

The aim was to build a new model, a standard model, based on the MLP. The method was performed as we have recently described [28, 29]. The input layer included the predictors (12 nodes). The number of hidden layers was automatically computed. The output was the lymphoma subtypes as FL, MCL, DLBCL, BL, and MZL. The neural network managed to predict several lymphoma subtypes with an overall percent correct of 79%. For lymphoma subtypes, the percentage was FL (85%), MCL (88%), DLBCL (79%), BL (80%), and MZL (44%). The characteristics of the network and the classification matrix are shown in Figure 3. Of note, MZL had a low predictive accuracy using as predictors the several genes. The same markers by immunohistochemistry at the protein level are being used by histopathologists to diagnose this lymphoma subtype. But it is not an easy diagnosis and, in some cases, the final diagnosis is “indolent” mature BCL unspecified.

**Figure 4:**
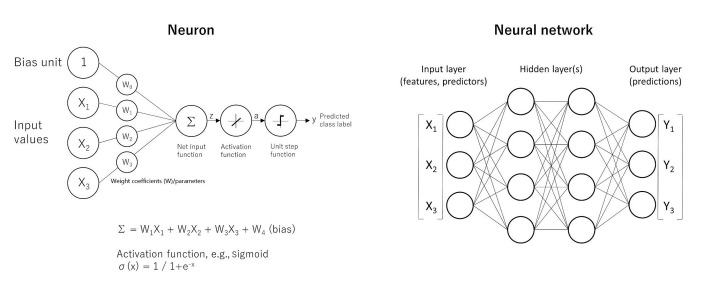
The basics of neural networks. Artificial neural networks have the ability of function approximation. Functions are “input-output” machines, in which a set of predictors (x) that are numbers are taken and an output (y) is created. The function is what defines the relationship between x and y. A simple form of neural network is the feed-forward network, also known as MLP. The MLP network is comprised of several units called neurons. The neurons take many inputs (x), but only produce one output (y). Each input is multiplied by its own weight (w), and in the equation one extra weight (bias) is added. After addition (Σ), the product is passed to an activation function to add nonlineality such as the sigmoid function (σ). The point is that each neuron is responsible for learning a small piece of the overall function. A MLP is a neural network. The architecture characterized by an input layer that contains the predictors, a hidden layer that contains unobservable nodes, and an output layer that contains the responses

Neural networks predict a target variable, which can be continuous or categorical, based on one or more predictors. Neural networks search for patterns in the data. The MLP is a feed-forward, supervised learning model [36]. The formulas are shown in Figure 4. Further information of MLP calculations is found in the following references [39–42].

## Conclusions

The classification of lymphoid neoplasms reflects a consensus among hematopathologists, geneticists, and clinicians on both updates to well-established subtypes and the addition of some new entities. Morphology, immunophenotype, clinical features, and molecular pathology analyses, such as next-generation sequencing are all incorporated into the classification. AI has advanced rapidly recently, and its role in medicine is becoming increasingly important. AI combines computer science and datasets to make predictions or classifications based on input data [27]. This paper showed several examples of the use of machine learning and neural networks to predict the prognosis and to classify mature B-cell neoplasms. Prediction of lymphoma subtypes based on conventional cell-of-origin markers was also calculated using a neural network. In the future, it is expected that AI will be incorporated into the classification as another bioinformatics tool to analyze complex data.

## Abbreviations

AI: artificial intelligence
BCLs: B-cell lymphomas
BL: Burkitt lymphoma
C & R: classification and regression
CASP8: caspase-8
DLBCL: diffuse large B-cell lymphoma
EBV: Epstein-Barr virus
ENO3: enolase 3
EZH2: enhancer of zeste 2 polycomb repressive complex 2 subunit
FL: follicular lymphoma
GCB: germinal center B
HBCL: high-grade B-cell lymphoma
HHV-8: human herpesvirus 8
HL: Hodgkin lymphoma
ICC: International Consensus Classification
Ig: immunoglobulin
IRF4: interferon regulatory factor 4
KSHV: Kaposi’s sarcoma-associated herpesvirus
LBCL: large B-cell lymphoma
LPL: lymphoplasmacytic lymphoma
MCL: mantle cell lymphoma
MYD88: myeloid differentiation primary response 88
MZL: marginal zone lymphoma
NOS: not otherwise specified
TNFAIP8: tumor necrosis factor-alpha-induced protein 8
Treg: regulatory T
TP53: tumor protein 53
WHO: World Health Organization

## Declarations

### Availability of data and materials

The datasets for this study are available upon request to Joaquim Carreras (joaquim.carreras@tokai.ac.jp).

### Copyright

© The Author(s) 2024.
